# Characterization of Si and SiO_2_ in Dust Emitted during Granite Polishing as a Function of Cutting Conditions

**DOI:** 10.3390/ma15113965

**Published:** 2022-06-02

**Authors:** Jules Kouam, Victor Songmene, Ali Bahloul, Agnes M. Samuel

**Affiliations:** 1 Department of Mechanical Engineering, École de Technologie Supérieure (ÉTS), 1100 Notre-Dame Street West, Montréal, QC H3C 1K3, Canada; jules.kouam@etsmtl.ca (J.K.); agnes-marie.samuel@etsmtl.ca (A.M.S.); 2 Institut de Recherche Robert-Sauvé en Santé et Sécurité du Travail (IRSST), Montréal, QC H3A 3C2, Canada; ali.bahloul@irsst.qc.ca

**Keywords:** granite polishing, machining conditions, crystalline silica, particle characterization, occupational safety

## Abstract

Particles emitted during manufacturing processes such as polishing can represent a serious danger for the environment and for occupational safety. The formation mechanisms responsible for these dust emissions include chip formation, friction at the tool/workpiece and chip/tool interfaces, shearing and cutting. These mechanisms thus depend on workpiece and tool properties, as well as the polishing conditions. In the case of granite polishing, particle emissions during polishing can contain chemical compounds such as silica, which represent harmful health risks for the worker. It is therefore important to characterize the particles emitted and to search for possible interactions between the particles (size and composition) and the machining conditions in order to find ways of reducing emissions at the source. In this study, an investigation was undertaken to characterize the particles emitted during granite polishing as a function of polishing conditions, type of granite, and abrasive grit sizes used. Scanning electron microscopy (SEM) was employed for particle morphology characterization and particle grain size and chemical composition were evaluated using X-ray diffraction (XRD) and energy dispersive X-ray (EDX) techniques, respectively. Results show that the influence of polishing speed and feed rate on particle emission depends mainly on the granite type used, providing useful information for controlling the polishing procedure, and thereby dust emission.

## 1. Introduction

Granite is an appreciated material because of its high mechanical properties such as wear resistance and good stability. It is also useful for house and building decorations, landscaping, and urban development, etc. 

Polishing is the machining operation most used for shaping granite compared to drilling, grinding, and saw milling. The aim of polishing is to grind the surface of the workpiece and produce the desired finished surface quality. During this process, the abrasive used scrapes the surface and generates chips and particles, where particle emissions could be compared to the removal of very small chips. Yilmaz et al. (2013) [1] studied the effect of cutting tools on chip thickness during machining of granite and reported that a sharp cutting tool produces larger chips than a worn tool.

According to Ling (1993) [2] and Li (1990) [3], after polishing, a thin ‘glossy film’ covers the surface of the granite workpiece. To learn more about it, Huang et al. (2002) [4] carried out an investigation to elucidate the appearance of this glossy film. However, no authors addressed the composition of the glossy film.

**Figure 1:**
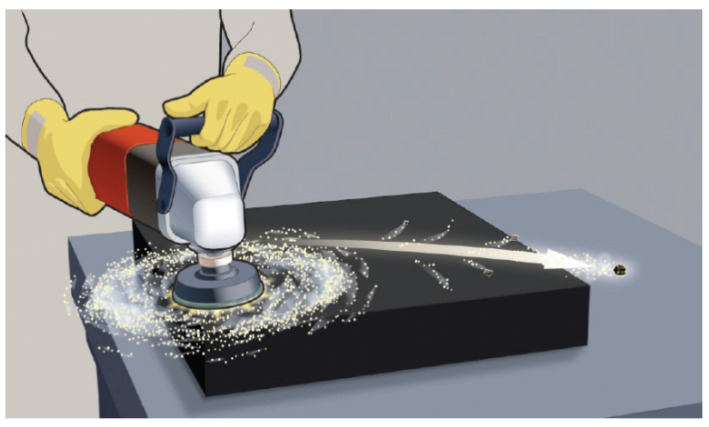
Schematic representation of particle dispersion when polishing granite (Goyer et al. (2010) [7]).

In the studies of Xie (2010) [5] and Xie and Tamaki (2007) [6], it was found that depending on the machining process applied, the type of abrasive used depended on the type of granite and could be susceptible to generating fine and ultrafine particles. Figure 1 presents a schematic representation of the particle dispersion when polishing granite [7].

Some researchers as Ahmad et al. (2011) [8] have studied the nano-toxicity of particle emissions during saw milling of granite and have found that nanoparticles are significantly more toxic than micro-particles. However, in their study, the chemical composition of the granite, which could be the source of this toxicity, was not investigated.

Pozzi et al. (2003) [9] have shown that simple inhalation of ultrafine particles (PM2.5) in urban air could pose a serious threat to human health. These particles are more harmful as they can penetrate the respiratory tract area (Chung (1996) [10]). 

Some occupational silicosis has been reported for people involved in granite manufacturing (Graham et al. (2004) [11] and Kulkarni (2007) [12]). In general, the potential risk associated with exposition to ultrafine particles is well documented (Pozzi et al. (2003) [9], Chung (1996) [10] and Seaton et al. (1995) [13]). 

Most of such research works were done on particles emitted during machining of metal, such as those of Sutherland et al. (2000) [14], Songmene et al. (2008) [15], Kouam et al. (2012) [16], and Khettabi et al. (2013) [17]. In these studies, however, the chemical composition of the dust was not investigated. 

In the particular case of granite, its grinding/polishing produces crystalline silica dust which, after inhalation for long periods, can cause alveolar inflammation and several respiratory diseases such as silicosis and lung cancer [18]. It has been recommended that granite manufacturers ensure that during a five-day work week (8 h/day), workers’ exposure to quartz does not exceed a level of 50 g/m^3^ (OSHA, 2016) [19].

For improving the occupational safety in granite transformation, different avenues are being explored, such as material substitution, process parameters and machining strategy modification [20], use of cutting fluids [21], dust caption [22] at the source for future use. Other researchers [23,24,25] have focused on using granite residues to incorporate them into materials such as concrete and mortar. In this context, De Azevdo et al. (2019) [23] investigated the feasibility of using granite residues obtained from cutting and polishing processes of granite blocks sourced from the city of Cachoeiro do Itapemirim in Brazil, as a partial substitute for fine aggregates in mortar. The granulometric characterization carried out by the authors has shown that both natural sand and granite residues are within the lower and upper range normalized in Brazil, aimed at providing adequate compaction to the cement matrix. The authors also analyzed the different oxides present in the granite residues.

Prokopski et al. (2020) [24] have shown that using granite powder in concrete mixes increases the workability, reduces porosity and increases the compressive strength of the concrete. Tangaramvong et al. (2021) [25] investigated the influence of granite particle wastes as replacement materials for natural river sand in concrete. They found no reduction in compressive strength of the new concrete with up to 50% granite particles, but the tensile strength was reduced, especially in the low-strength concrete where the granite wastes replaced natural sand in the concrete in higher proportions. The setting time of high-strength concrete reduced with the amount of granite wastes higher than 30%.

These authors ([23,24,25]), however, did not link the granite dust morphology, composition or distribution with the mechanical granite transformation process parameters.

Very limited articles have been published on particle emissions when polishing granite. Kouam et al. (2013) [26] studied particle emissions during belt polishing of granite. The results of their investigation showed that the type of granite, the machining parameters, and the method of machining have an impact on the particle emissions. Thereafter, rotational, dispersion, MQL (minimum quantity lubricant), and tool path polishing were investigated by these researchers (Saidi et al. (2015) [27], Songmene et al. (2018) [20,21] and Saidi et al. (2018) [28]). Despite the health hazards associated with granite polishing, there appear to be only a few studies on silica dust characterization, those of Carrieri et al. (2020) [29], Ramkissoon et al. (2022) [30], and Hall et al. (2022) [31], particularly in light of increasing cases of accelerated silicosis related to the use of engineered (artificial) stones in the construction industry.

In view of the above, the main aim of this research study is to characterize the dust emitted during rotational polishing of granite in relation to the abrasives and polishing conditions used; the goal being to suggest ways of reducing such emissions at the source. This article is part of a global research work characterizing particles and dust emission when polishing granites materials [22]. The aim of this global work was to determine ways of eliminating and reducing at source the fine and ultrafine dusts containing crystalline silica that are emitted during the processing of granite in order to better protect the health and safety of workers. Among other conclusions, it was shown that the size of the abrasive disc and the polishing conditions have a significant effect on the emission of fine particles (FP) and ultrafine particles (UPF) as well as on the distribution of the emitted dust in the space surrounding this process. In addition, the analysis of the morphology of the FP and UPF showed that the silica content increases with the size of the particles emitted by the granite polishing process.

## 2. Materials and Methods

The experimental procedure steps for this study were carried out as follows.

- Reception of workpieces from A. Lacroix Granit, Saint-Sébastien, QC, Canada;
- SEM and petrographic characterization of samples;
- Sample preparation (polishing using grit 45);
- Experimental tests using different polishing speeds and feed rates;
- Collection of polishing granite residues, using filters installed in TSI DustTrak Aerosol Monitor instrument;
- SEM characterization of granite residues;
- EDX characterization of granite residues;
- X-ray diffraction analysis of granite residues to compare phases grain sizes.

Dry polishing tests were carried out on a white granite and on a black granite (Canadian anorthosite) whose chemical compositions are shown in Table 1. The composition of granite comprises 70–77% silica (SiO_2_), 11–13% alumina (Al_2_O_3_), 3–5% potash (K_2_O), 3–5% soda (Na_2_O), 1% lime (CaO), 2–3% total iron (FeO and Fe_2_O_3_), and less than 1% magnesia (MgO) and titania (TiO_2_). The minerals quartz and feldspar always present in granite generally give granite a light color, ranging from pinkish to white. As opposed to white granite, black granites are dark colored igneous rocks, usually gabbro, and their chemical and mineralogical compositions are quite different from that of granites. Their densities, hardness (6–7 on the Mohs scale) and compressive strengths (200–225 MPa) being similar, however, these stones can be used satisfactorily for the same purposes in industry. With respect to grain size, granite is coarse grained with grain size ranging from 1 to 25 mm, while black granite (gabbro) has a grain size of 1 mm or more. 

**Figure A1:**
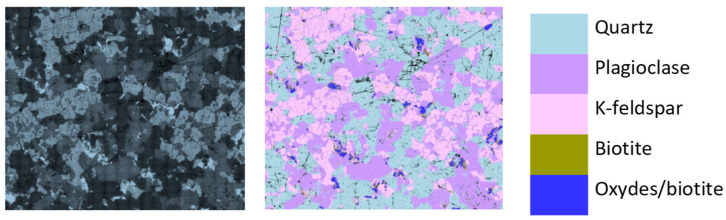
White granite: scattered electrons image (OIS Service Géoscientifiques Inc., Chicoutimi, QC, Canada).

**Figure A2:**
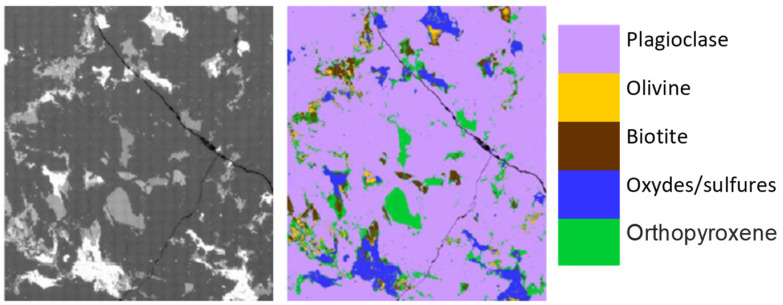
Black granite (Canadian anorthosite): scattered electrons image (OIS Service Géoscientifiques Inc., Chicoutimi, QC, Canada).

The workpiece blocks used in the present study were provided by the company A. Lacroix Granit in Saint-Sébastien, Quebec, Canada. In a previous study, Haithem et al. (2021) [26] presented the properties of the white and black granite workpieces used by them together with the mineralogical analysis: Black granite (0% quartz; 83% plagioclase) and white granite (41% quartz; 32% plagioclase and 23% K-felsdpar). The same two types of granites/stones have been used in this study. The average density of the white and black granites are 2.7 g/cm^3^ and 3.1 g/cm^3^, respectively. Figure A1 and Figure A2 and Table A1 in the Appendix A provide the minerals present in the two granite types used and their grain sizes and proportions. 

Petrographic and scanning electron microscopy analysis performed by IOS Geoscientifiques Inc. (Chicoutimi, QC, Canada) revealed that the black granite sample, consisting of several centimeter-sized polished plate fragments of 2.5 cm thick, is an anorthosite, whose composition is at the limit of the leucoferronorite field, due to the abundance of orthopyroxene (21%) in its composition. Indeed, the rock is black, massive, with coarse grains mainly made up of plagioclase. There are also iron and titanium oxides and sulfides (pyrrothite, chalcopyrite). It consists of 67% plagioclase, 21% orthopyroxene, 4% biotite, 2% olivine more or less altered, 4.5% iron-titanium oxides. It does not contain quartz. 

The white granite sample, on the contrary, is a medium to coarse grained granite with an automorphic granular and porphyritic texture. SEM imaging indicated that it contains 41% quartz. From here on in this article, the two stones will be referred to as white granite and Canadian anorthosite.

It is observed from Table 1 that the white granite contains more silicon (Si) in weight (about 52 wt%) as compared to the Canadian anorthosite (11 wt%) in the workpiece.

The machining unit used is a Computer Numerical Control (CNC) milling machine. The following dry polishing conditions and parameters were used:

- Polishing speeds: 250–2500 rpm
- Feed rate: 254–2032 mm/min
- Pressure: 0.2 bar
- Abrasive grit: 45, 60 and 100

**Figure 2:**
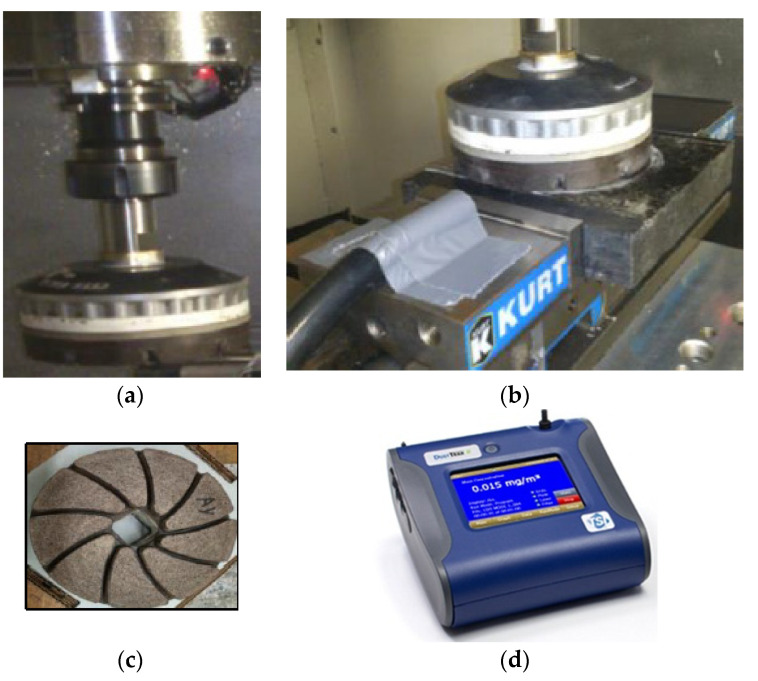
Experimental setup and equipment used in the present study (Saidi et al. (2018) [22]). (**a**) Polishing tool; (**b**) Particle sampling position; (**c**) Abrasive tool geometry; (**d**) DustTrak Monitor for particle collection.

A pressure of 0.2 bar was selected to simulate human effort expended in the industrial environment. The particles were collected directly inside the polishing machine using a TSI DustTrak Aerosol Monitor instrument (Model 8530), equipped with a 2.5 μm impactor (Figure 2) and a cap to collect particles for subsequent characterization. The sampling pump flow rate was set at 1.5 L/min. 

X-ray diffraction (XRD) patterns were obtained using an XRD diffractometer (PANalytical X’Pert Pro Materials Research Diffractometer) with CuK_α1_ (λ_Kα1_ = 1.5406Å) and CuK_α2_ (λ_kα2_ = 1.5444Å) radiation, which was used to evaluate the grain size of the particles obtained during dry machining under different polishing conditions. 

Scanning electron microscopy (SEM) employing a Hitachi 3600 instrument was used for examining the particle morphology, and an energy dispersive X-ray spectroscopy (EDS) unit associated with the SEM was used for analysis of the particle chemical composition. 

## 3. Results and Discussion

### 3.1. Analysis of Si and SiO_2_ Particle Grain Size Using X-ray Diffraction (XRD)

The XRD patterns of the particles emitted during polishing were analyzed according to the deconvolution of the peak in two Lorentzians corresponding to the two components λ_Kα1_ and λ_Kα2_ of the wavelength λ_Kα_ of copper. This procedure yielded a measurement of full width of the peak at half maximum (FWHM) for each peak, corresponding to a well-defined wavelength, and was applied to the whole pattern. The details of this procedure have been already reported by Kouam et al. (2008) [32] and Kouam et al. (2013) [26] previously.

From the fits to the experimental peaks, it is possible to determine the parameter *ω* of the diffraction peaks and to compute the average grain size d*_m_*, using the relation (1) proposed by Scherrer:

(1) $$d_{m}=\frac{k.\lambda }{\omega \cos\theta_{hkl}}$$

where *k* is a constant which depends on the form of the crystallites and on the Miller indices. Bragg (1949) [33] showed that in many cases, the value of *k* is close to 0.9; *θ_hkl_* is the diffraction angle, *λ* is the wavelength of the incidental radiation, and *ω* corresponds to the FWHM. It must be noted here that only the Si and SiO_2_ peak diffraction positions were only considered.

**Figure 3:**
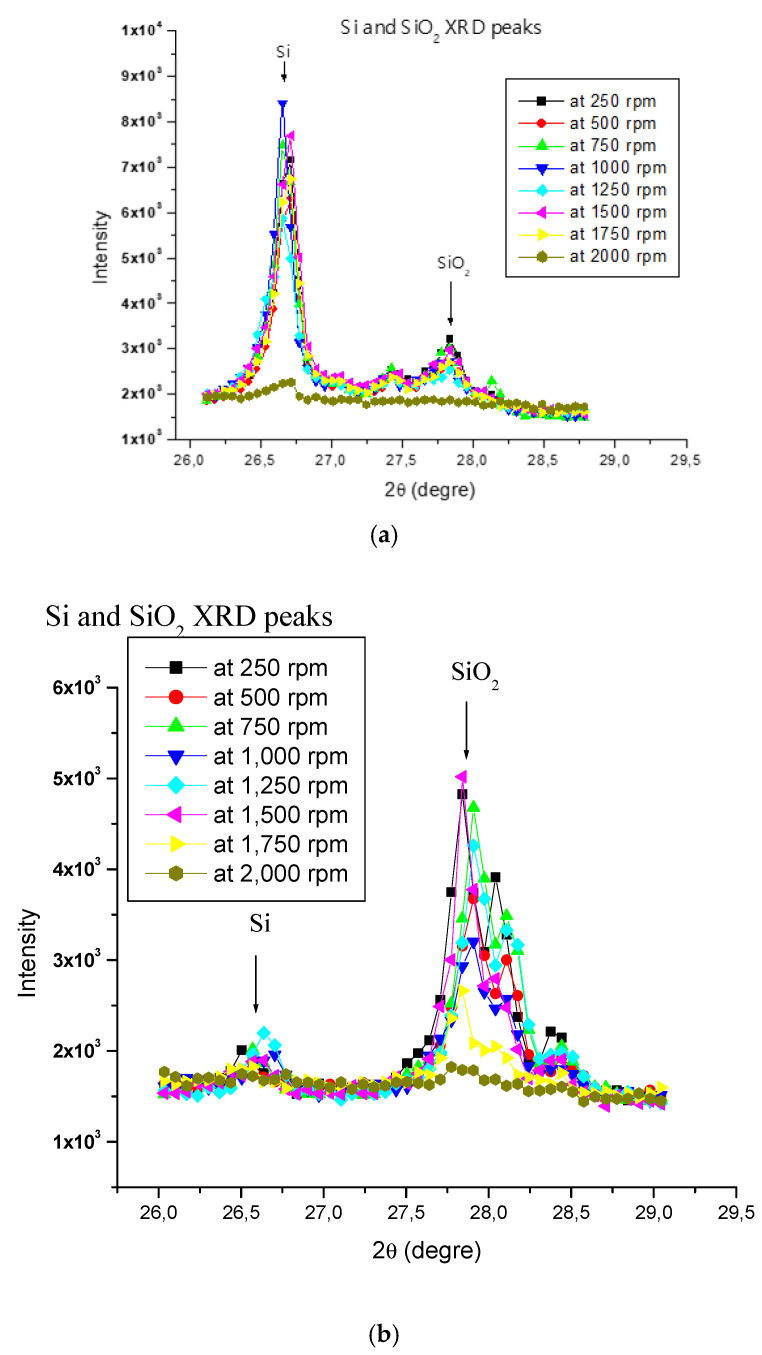
XRD patterns obtained at different polishing rotation speeds for (**a**) white granite, and (**b**) Black granite (Canadian anorthosite), using 508 mm/min feed rate, 0.2 bar pressure, and 60 grit abrasive.

**Figure 4:**
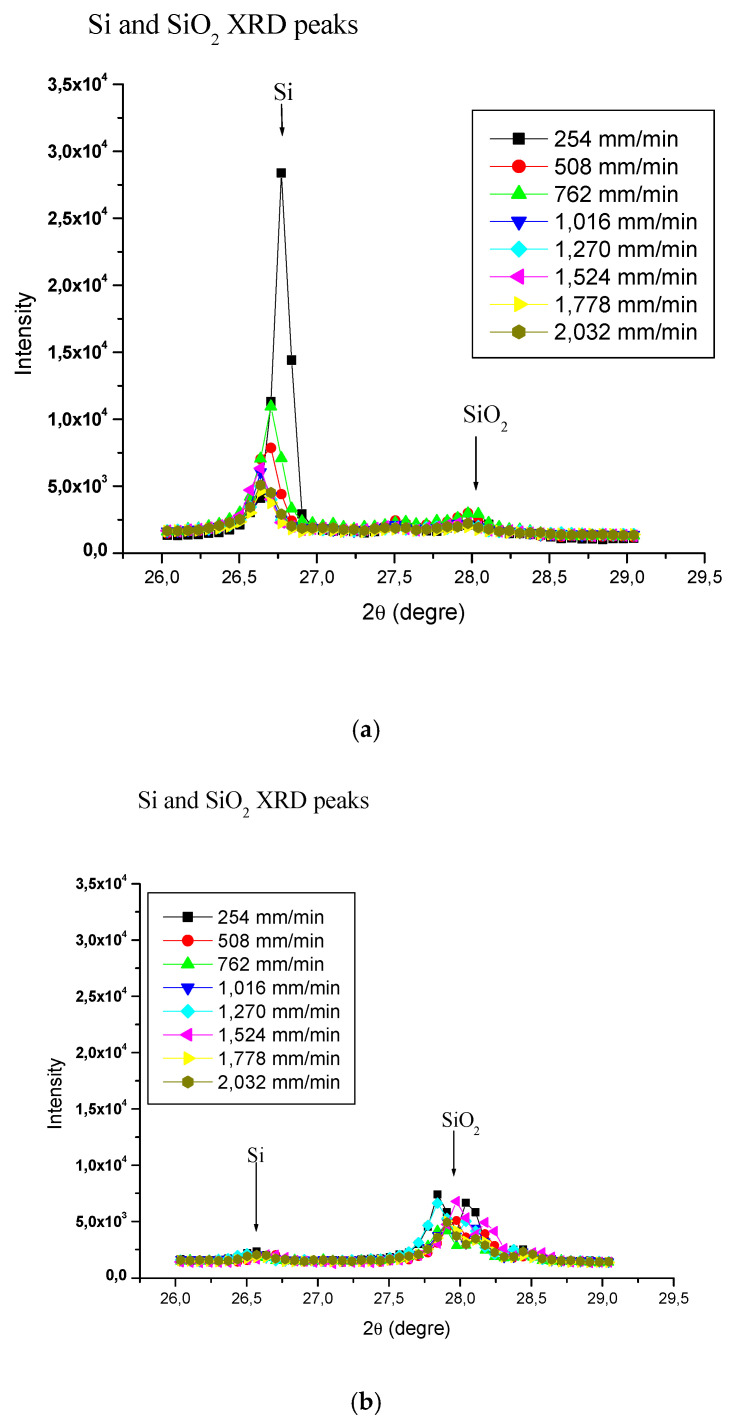
XRD patterns obtained at different feed rates for (**a**) white granite, and (**b**) Black granite (Canadian anorthosite), using 1000 rpm polishing rotation speed, 0.2 bar pressure and 60 grit abrasive.

Figure 3 and Figure 4 present the XRD patterns of particles respectively collected at different polishing speeds and feed rates for the white granite and the Canadian anorthosite stones, respectively.

Certain observations may be noted from the two figures as follows:

- The peak intensity of Si is high in the white granite as compared to the Canadian anorthosite while that for SiO_2_ is low in the white granite compared to the Canadian anorthosite. This general observation could be explained by the initial Si content of the granite workpiece, which is higher in the white granite than in the Canadian anorthosite. If we consider the mineralogy of the two stones (presented in Supplementary Materials), it is seen that white granite contains about 42% quartz or SiO_2_ with a Mohs hardness of 7), whereas the Canadian anorthosite contains no quartz at all, but 67% plagioclase, which has the formula NaAlSi_3_O_8_–CaAl_2_Si_2_O_8_ and is a continuous solid solution series, categorized more properly as the plagioclase feldspar series within the feldspar group. It has a hardness of 6 to 6.5 on the Mohs hardness scale. In addition, white granite also contains 38% K-feldspar and 19% plagioclase. The mineral feldspar contains mainly Al, Si, and O atoms (with the formula KAlSi_3_O_8_–NaAlSi_3_O_8_–CaAl_2_Si_2_O_8_, Mohs hardness 6–6.5) whereas quartz contains Si and O atoms. Thus, the major chemical element present in quartz is Si, whereas in feldspar it is Al. Taking all these points into consideration, it is expected that the Si peak will have a much higher intensity in the white granite XRD pattern, compared to the Canadian anorthosite.

In comparison, the peaks for SiO_2_ display much lower intensities at all polishing rotation speeds for the white granite (between 2500 and 3500), whereas they are relatively higher (2700–5000) in the Canadian anorthosite. This may result from the differences in the hardness and the abrasion coefficients of quartz, plagioclase and feldspar constituting the two granite types. The aggregate abrasion value (AAV) or the resistance to abrasion is 2.6 for granite (i.e., white granite) and 4.1 for gabbro (i.e., Canadian anorthosite). The smaller the AAV value, the more resistant the material is to abrasion.

- The peak diffraction intensities of Si and SiO_2_ are low (Figure 3a) as compared to the peak intensities of Si and SiO_2_ observed in Figure 4a for white granite with variation in feed rate, at a polishing speed of 1000 rpm. Similar observations are noted for the Canadian anorthosite (Figure 3b and Figure 4b), with the intensities much weaker in this case. These results indicate that the feed rate has a greater influence on the peak intensities than the polishing speed.

**Figure 5:**
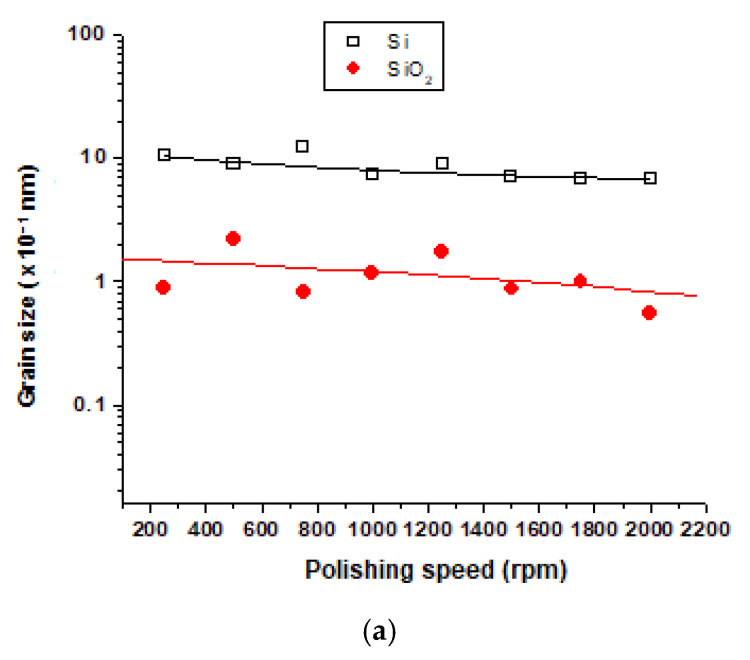

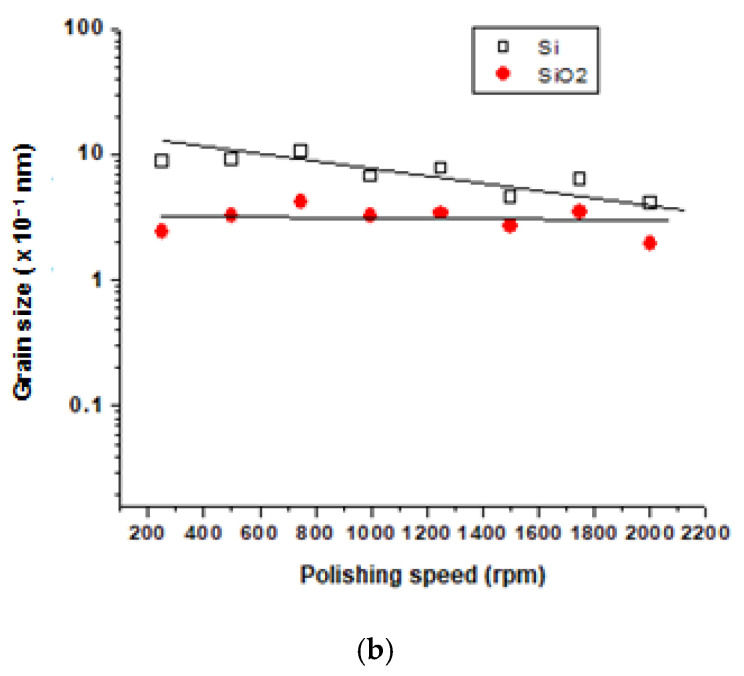
Grain size of Si and SiO_2_ particles at different polishing speeds for (**a**) white granite, and (**b**) Black granite (Canadian anorthosite), using 508 mm/min feed rate, 0.2 bar pressure, and 60 grit abrasive.

**Figure 6:**
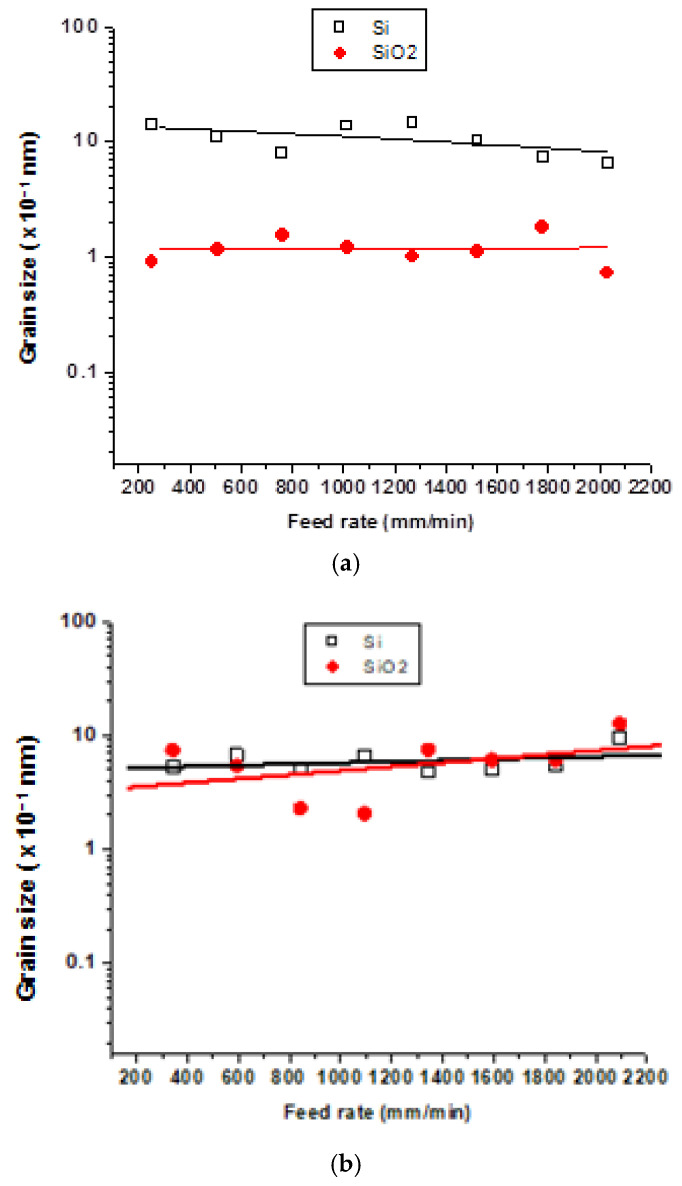
Grain size of Si and SiO_2_ particles emitted at different feed rates for (**a**) white, and (**b**) Black granite (Canadian anorthosite) using 1000 rpm polishing rotation speed, 0.2 bar pressure and 60 grit abrasive.

Figure 5 and Figure 6 present the grain size data i.e., the *d_m_* values for Si and SiO_2_ particles emitted during polishing of white and Canadian anorthosite at different polishing rotation speeds and feed rates, respectively, obtained using Equation (1) proposed by Scherrer. 

The main observation in Figure 5 is that the grain size in Si particles is higher compared to SiO_2_. Moreover, it decreases slightly with an increase in the polishing speed used in the case of white granite (Figure 5a) as compared to the Canadian anorthosite (Figure 5b). This may be due to the higher concentration of Si in the white granite workpiece compared to the Canadian anorthosite (Kouam et al. (2013) [26]). The presence of the accessory mineral biotite (K(Mg,Fe)_3_(AlSi_3_O_10_)(F,OH)_2_) in the two granites (see Appendix A) may also affect the Si concentration, in that the refractory nature of this mineral would make it difficult to polish, giving smaller particles. It is also observed in Figure 5a (white granite) that the SiO_2_ grain size decreases as the polishing speed increases. For the Canadian anorthosite, however, Figure 5b shows that in this case the Si particle grain size decreases more rapidly as compared to SiO_2_ with the increase in polishing speed, probably for the same reason, i.e., due to the presence of biotite in the Canadian anorthosite also. These data were obtained using a feed rate of 508 mm/min. 

Figure 6 presents the grain size data for Si and SiO_2_ particles emitted during polishing at different feed rates. In the case of white granite, Figure 6a shows similar observations as those noted in Figure 5a, namely, that in the case of feed rate also, the grain size of the Si particles is higher than that of SiO_2_, which could be attributed to the higher concentration of Si in the white granite workpiece as mentioned previously [26]. It is also observed in Figure 6a (white granite) that the Si grain size decreases as the feed rate increases. Thus, the cutting speed and feed rate have the same influence when polishing white granite. In the case of Canadian anorthosite (Figure 6b), however, apparently there is no influence of the feed rate when polishing is carried out at 1000 rpm and beyond. It is difficult to provide an exact reason for this, except to suggest that a combination of the composition, processing parameters and particle measurement technique would affect the result obtained. In this connection it is worth mentioning the work of Hall et al. (2022) [31], who characterized and compared dust emissions and respirable crystalline silica (RCS) in natural and artificial stones, using XRD and scanning electron microscopy, where a wide range aerosol spectrometer was used to measure the particle size distribution. They reported that the higher the silica content in the original stone, the higher the level of silica found in the dust emission during processing of the stone.

**Figure 7:**
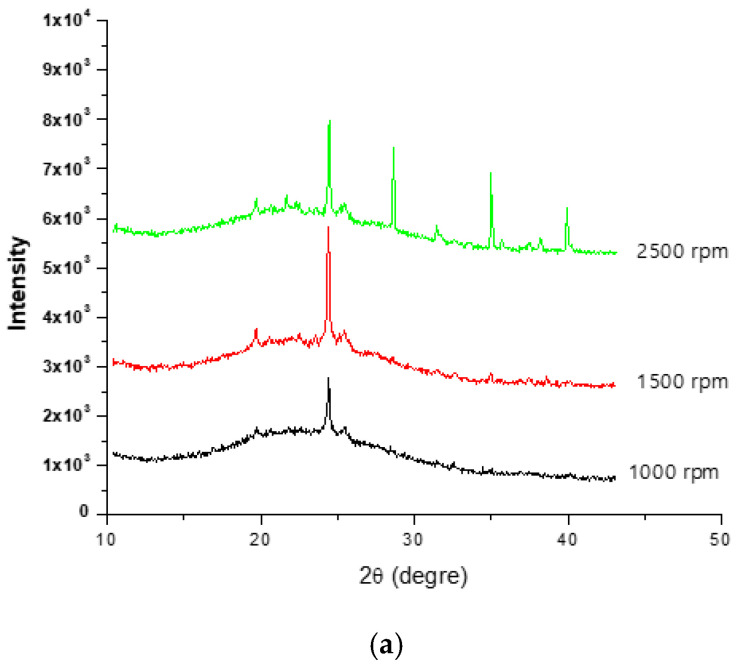

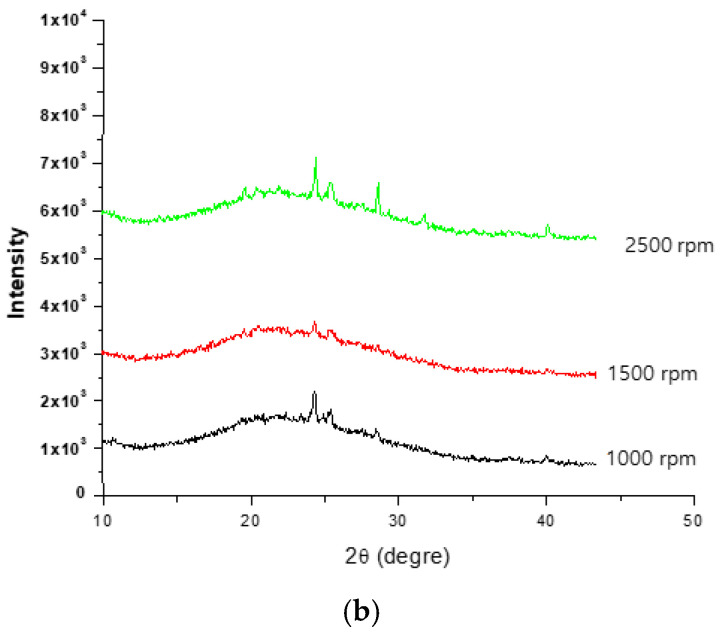
XRD spectra obtained using (**a**) 45 grid abrasive, and (**b**) 100 grid abrasive at different polishing speeds for white granite.

Figure 7a,b present the XRD patterns of particles collected using 45 grit abrasive and 100 grit abrasives, respectively, using polishing speeds of 1000, 1500, and 2500 rpm on white granite. 

Similar XRD patterns were obtained for Canadian anorthosite. It is observed that the peak intensities at the same polishing speeds using the 45 grit abrasive (Figure 7a) are high compared to those obtained using the 100 grit abrasive (Figure 7b). This difference could be attributed to the fact that the use of a 45 grit abrasive produces more particles compared to the use of a finer i.e., 100 grit abrasive (Saidi et al. (2015) [27]). Thus, as Figure 7 shows, particle emission would depend not only on the abrasive grit type/size used, but also on the polishing conditions. However, the mineralogical composition would have an important influence on these results, as the constituent minerals would react differently to abrasion during the polishing process.

### 3.2. Qualitative Analysis of Emitted Particles Using Scanning Electron Microscopy (SEM) 

The results presented in Section 3.1 have shown that the particles emitted during polishing depend not only on the properties of the workpiece material, but are also affected by the polishing conditions, including the polishing speed and feed rate, and also by the grit size of the abrasive used in the polishing of the granite workpieces investigated in this study. Thus, a change in polishing parameters (polishing speed and feed rate) would be expected to affect the morphology of the particles emitted and possibly their agglomeration. Characteristics of particles emitted during polishing of the white and Canadian anorthosite workpieces were examined using a Hitachi 3600 scanning electron microscope, operating at 75 mA current and 15 kV.

**Figure 8:**
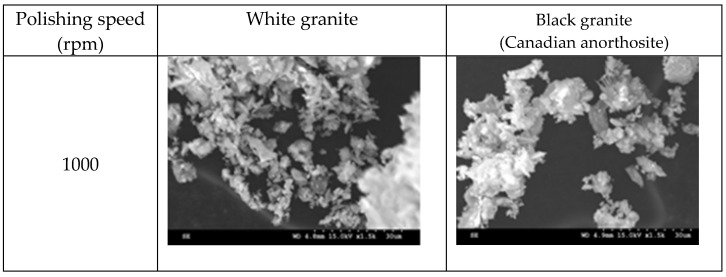

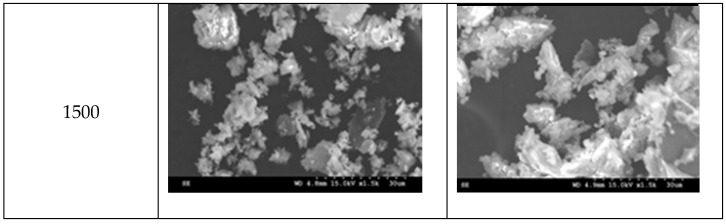
SEM images of particles obtained when polishing white and Canadian anorthosite at different speeds (feed rate—508 mm/min; grit size—60; pressure—0.2 bar).

Figure 8 displays electron images showing the morphologies of the particles obtained when polishing white and Canadian anorthosite at two different polishing speeds using 60 grit abrasive and a feed rate of 508 mm/min.

The general observation is that the particles appear in elongated form. This morphology could be the result of the tool displacement during the polishing process. At the lower polishing speed, the particle extension is more pronounced and decreases with the increase in the polishing speed (to 1500 rpm) for the white granite compared to the Canadian anorthosite. It is also to be noted that particle agglomeration is greater with the increase in polishing speed for the Canadian anorthosite compared to the white granite. This observation could result from the difference in their mechanical properties as well as the high Si content of the white granite compared to the Canadian anorthosite. As mentioned earlier in the context of Figure 3 and Figure 4, the differences in the hardness and the abrasion coefficients of quartz, plagioclase, and feldspar constituting the two granite types, could also affect these results. The aggregate abrasion value (AAV) or the resistance to abrasion is 2.6 for white granite and 4.1 for the Canadian anorthosite, so that the white granite with its smaller AAV value would be more resistant to abrasion. This would influence the particle morphology depending on the processing conditions.

**Figure 9:**
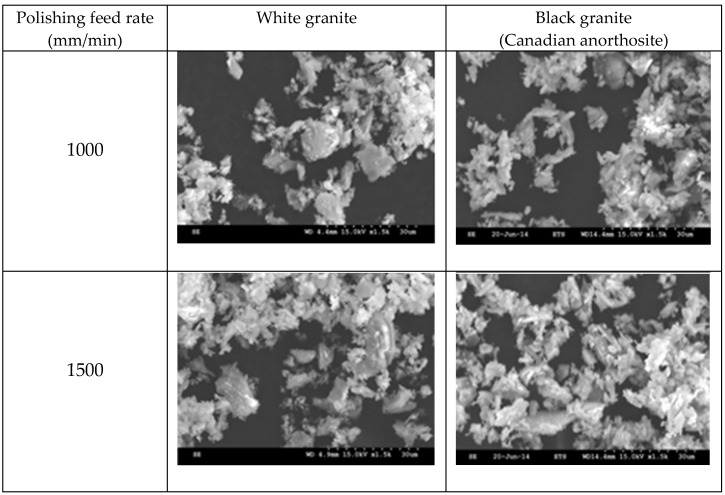
SEM images of particles obtained when polishing white granite and Canadian anorthosite at different feed rates (polishing speed—1000 rpm, grit size—60; pressure—0.2 bar).

Figure 9 presents electron images of the morphologies of the particles obtained when polishing white granite and Canadian anorthosite at two different feed rates using 60 grit abrasive at 1000 rpm polishing speed and 0.2 bar pressure. 

As was observed in Figure 8, when varying the feed rate (at a polishing speed of 1000 rpm), the particles emitted also present an elongated form as shown in Figure 9. The same explanation of surface renewal applies in this case also, and explains why particles are not crushed. It is also seen that the particle morphology is similar at different feed rates for both granite types. The agglomeration, however, is more pronounced at different feed rates (Figure 9) compared to that obtained at different polishing speeds (Figure 8). 

Ramkissoon et al. [30] studied the characterization of dust emission generated by dry cutting of engineered stones and natural stones including white granite, black granite and marble, using SEM analysis. The α-quartz (or silica) contents were respectively 30.1% and 3.5% for black and white quartz whereas the particle sizes were 503 ± 11 and 634 ± 22 nm. As reported by Haithem et al. [34], the SEM analysis of black and white granite used in our study showed that the black granite (Canadian anorthosite) contained no quartz but 83% plagioclase (another form of silica), while the white granite contained 41% quartz as well as plagioclase and K-feldspar. The particle sizes obtained in the present study were 0.12–1.5 nm for the Canadian anorthosite, and around 0.1 nm for the white granite workpieces. The disparity in particle sizes could result from the differences in both the means of generation and the method of collection of the emitted dust particles.

### 3.3. Composition of Emitted Particles Using Energy Dispersive X-ray (EDX) Analysis 

The chemical composition of the particles emitted during polishing processes depends not only on the workpiece material properties but also on the polishing conditions, and the grid abrasive used. As presented in Table 1, the tested granites contain different chemical elements; therefore, it is expected that the particles collected during polishing will be affected by the difference in composition of the workpiece materials, as well as by the other factors. 

**Figure 10:**
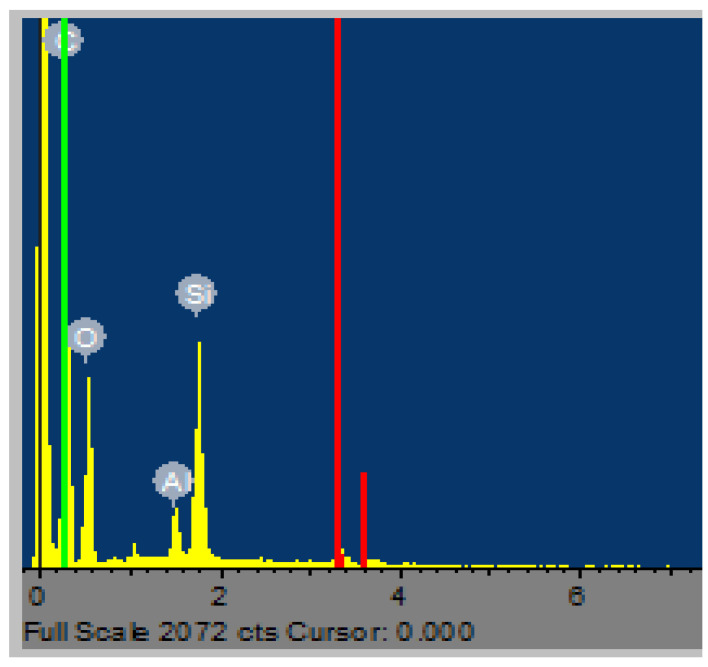
EDX spectrum of particles during polishing of granite (polishing speed—1000 rpm; feed rate—508 mm/min; abrasive grit size—60; pressure—0.2 bar).

Figure 10 shows an example of the EDX spectrum of particles obtained during polishing of white granite, showing the presence of different chemical elements Si, Al, O, and C.

**Figure 11:**
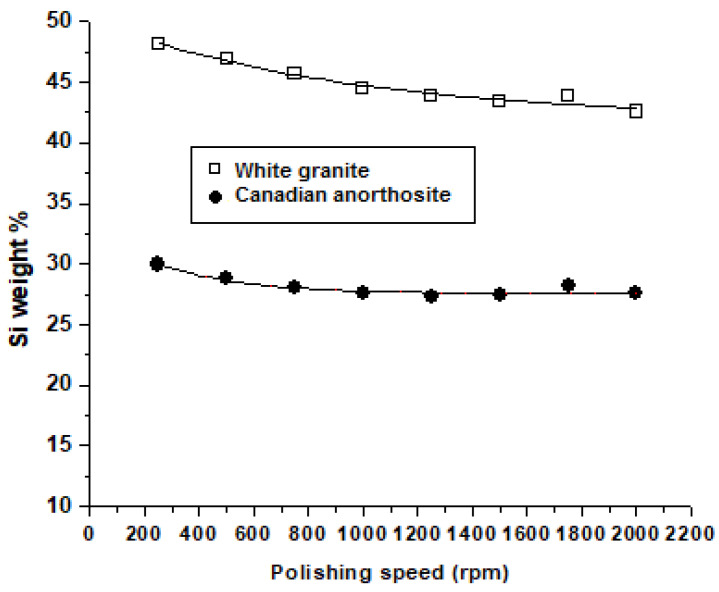
Variation of Si weight percent in emitted particles at different polishing speeds for the tested stones (feed rate—508 mm/min; grid size—60; pressure—0.2 bar).

**Figure 12:**
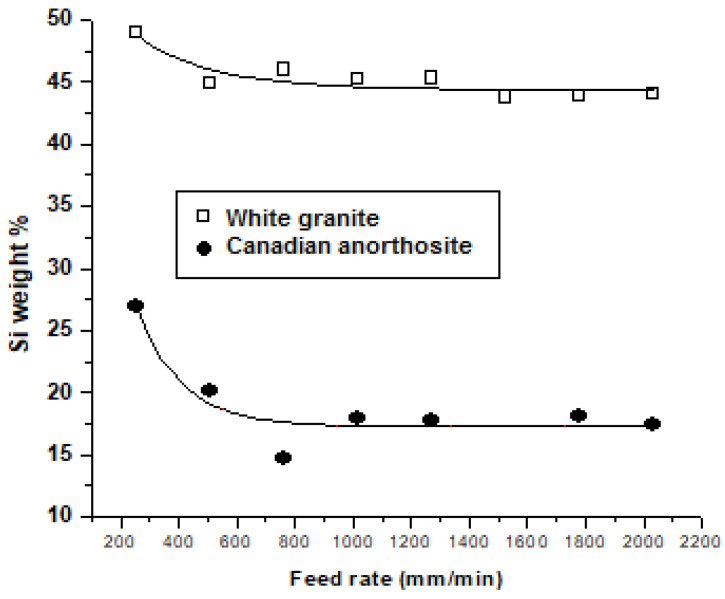
Variation of Si weight percent in emitted particles at different feed rates for the tested stones (polishing speed—1000 rpm; grid size—60; pressure—0.2 bar).

Figure 11 and Figure 12 present the Si content (wt.%) of the particles emitted at different polishing speeds and feed rates, respectively, during polishing of white granite and Canadian anorthosite.

From Figure 11, it may be seen that the Si weight percent decreases gradually with increase in the polishing rotation speed for both granite types. The same result is noted with the increase in feed rate (Figure 12) for the white granite, whereas in the case of the Canadian anorthosite, the Si content drops more rapidly in comparison, during the increase in feed rate up to 762 mm/min and then remains more or less stable as the feed rate increases to 1016 mm/min and beyond. It is also observed that the Si content is high when polishing white granite compared to Canadian anorthosite, on account of the higher concentration of Si in the white granite workpiece compared to the Canadian anorthosite workpiece [18]. Similar to the white granite, in the case of the Canadian anorthosite, the Si content also remains more or less stable as the polishing speed increases to 1000 rpm and beyond. 

## 4. Conclusions

This study was undertaken to characterize the dust emitted during rotational polishing of granite in relation to the abrasives and polishing conditions used, to determine ways of reducing such emissions at the source. The particles emitted during polishing were characterized using XRD, SEM, and EDX techniques. From the results obtained, the following may be concluded.

1. The grain size, morphology, and chemical composition of particles emitted during polishing of granite depend on the type of material, namely white granite versus Canadian anorthosite, the mineralogy, the abrasion coefficients of the constituent minerals, the polishing conditions (polishing speed and feed rate), and the abrasive tool used in the polishing process.
2. XRD analysis reveals that the peak diffraction intensity of Si is high, while that of SiO_2_ is low in white granite and vice versa in Canadian anorthosite due to the higher Si content of white granite (cf. 92% vs. 11%). Feed rate has a greater influence on the peak intensities than the polishing speed.
3. Polishing speed and feed rate have the same influence when polishing white granite, in that the grain size of emitted Si particles is higher than that of SiO_2_ particles, and the grain size of both particle types decreases with increase in polishing speed or feed rate. For the Canadian anorthosite, with increase in polishing speed, the Si particle grain size decreases more rapidly compared to SiO_2_, while there is no influence on the increase in feed rate. This information could prove useful in controlling the grain size of the particles emitted during polishing.
4. SEM images of emitted particles show that particles have an elongated morphology. Taking into account the sizes of the chips and particles emitted during the polishing process, we think that this is a consequence of the polishing (abrasive) action. Particle agglomeration is also observed, and increases with increase in polishing speed for the Canadian anorthosite compared to the white granite. While particle morphology is similar at different feed rates for both granite types, particle agglomeration is more pronounced at different feed rates compared to that at different polishing speeds.
5. EDX investigations show that the Si content (wt%) of particles emitted depends on the polishing conditions and on the grit size of the abrasive used for polishing. A relationship was proposed to predict the Si wt% as a function of the feed rate during polishing. Increasing the polishing speed and feed rate both reduce the Si content.

In summary, therefore, starting from the mineralogy of the workpiece material, the polishing speed, and feed rate could be selected in order to reduce the dust emission and silica content during polishing of the granite or anorthosite used. This would help to improve the level of environmental and occupational safety for workers in the industry. 

## Figures and Tables

**Table 1:** Chemical composition from EDX of the two tested stones (Kouam et al. [26]).

White granite	Black granite (Canadian anorthosite)
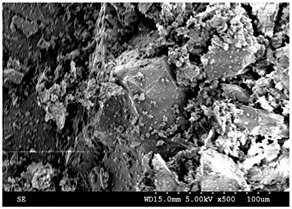	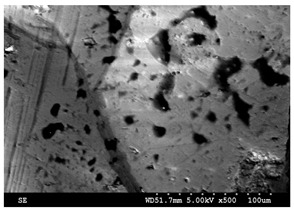
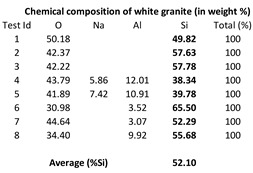	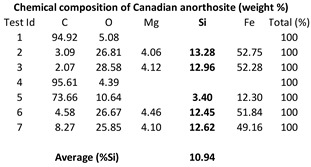

## Data Availability

Data available on request due to privacy or ethical restrictions.

## Supplementary Materials

The following supporting information can be downloaded at: https://www.mdpi.com/article/10.3390/ma15113965/s1, Table S1: Mineralogy and Grain Size of White Granite; Table S2: Mineralogy and Grain Size of Canadian anorthosite.

## Appendix A. Mineralogical Data for the Granites Used in This Study

Figure A1 and Figure A2 present the mineralogy of the white granite and Canadian anorthosite used in this study, while Table A1 compares the minerals present in the two granites and their proportions. The analysis was carried out by IOS Geoscientifiques Inc. in Chicoutimi, QC, Canada.

Table A1 compares the minerals present in the two granites and their proportions.

**Table A1:** Mineral distribution in white granite and Canadian anorthosite.

White Granite		Canadian Anorthosite	
Mineral	Proportion (%) ± 2%	Mineral	Proportion (%) ± 2%
Quartz	41.38	Quartz	0
Plagioclase	32.39	Plagioclase	83.61
Feldspar-K	23.14	Olivine	1.51
Biotite	1.14	Biotite	2.83
Oxides/biotite	1.95	Oxides/sulphides	5.20
		Orthopyroxene	6.85
