# Supplementary material for: Characterization of Si and SiO2 in Dust Emitted during Granite Polishing as a Function of Cutting Conditions
Source: Materials (Basel). 2022 Jun 2;15(11):3965. doi: 10.3390/ma15113965 (PMC9182374; doi:10.3390/ma15113965)
Supplement: Supplementary file 1 [file materials-15-03965-s001.zip › materials-1700591-supplementary.pdf]

Supplementary Material

Tables S1and S2 present the mineralogy of the white granite and Canadian anorthosite used in this study and the grain sizes of the mineral constituents. The analysis was carried out by IOS Geoscientifiques Inc. in Chicoutimi, QC, Canada.

Table S1 Mineralogy and Grain Size of White Granite

Mineral	%	Grain size
Quartz	40	1-5.5 mm
K-feldspar	38	0.5-7 mm
Plagioclase	19	1-6 mm
Albite	≤2	0.1-0.35 mm
Biotite	2	0.4-2 mm
Chlorite	tr	0.5 mm
Zircon	tr	≤0.12 mm
Apatite	tr	≤0.15 mm
Allanite?	tr	0.02-0.4 mm
Oxides of Fe and/or Ti	0.3	0.08-0.4 mm
Sulfides	tr	0.2 mm

Table S2 Mineralogy and Grain Size of Canadian anorthosite

Mineral	%	Grain size
Plagioclase	67	0.2-17 mm
Orthopyroxene 1 (Ferrosilicate)	21	1-10 mm
Biotite	<4	0.2-2 mm
Olivine (fayalite)	1	0.6-4 mm
Chlorite	1	-
Orthopyroxene 2 (hypersthene)	tr	3 mm
Clinopyroxene (diopside)	tr	35 um
Amphibole (hornblende)	tr	0.1 mm
Apatite	1	1-3 mm
Oxides of Fe and/or Ti	4.5	0.8-4 mm
Sulfides	tr	<0.3 mm
